# Engineering NK Cells for CAR Therapy—Recent Advances in Gene Transfer Methodology

**DOI:** 10.3389/fimmu.2020.611163

**Published:** 2021-01-07

**Authors:** Paula Schmidt, Martin J. Raftery, Gabriele Pecher

**Affiliations:** Medical Clinic of Hematology, Oncology and Tumor Immunology, CCM, Charité - Universitätsmedizin Berlin, Berlin, Germany

**Keywords:** natural killer cells, chimeric antigen receptor, gene engineering, transduce, electroporation

## Abstract

The development of chimeric antigen receptor (CAR) T cell therapy has introduced a new and effective strategy to guide and promote the immune response against tumors in the clinic. More recently, in an attempt to enhance its utility, this method has been expanded to novel cell types. One of the more successful variants has proven to be the expression of CARs in Natural Killer (NK) cells (CAR-NK). Gene engineering NK cells to express an exogenous CAR receptor allows the innate anti-tumor ability of NK cells to be harnessed and directed against a target tumor antigen. In addition, the biology of NK cells allows the development of an allogeneic cell therapeutic product useable with most or all patient haplotypes. NK cells cause little or no graft versus host disease (GvHD) and are therefore suitable for development of an “off the shelf” therapeutic product. Initial trials have also shown that CAR-NK cells rarely cause cytokine release syndrome. However, despite their potential NK cells have proven to be difficult to engineer, with high sensitivity to apoptosis and low levels of gene expression. The creation of optimized methods to introduce genes into NK cells will promote the widespread application of CAR-NK in research laboratories and the clinics.

## Introduction

NK cells are a subpopulation of lymphocytes central to the innate immune system and the innate response to viruses. In peripheral blood, ~10% of mononuclear cells are NK cells and are thus readily isolated from density gradient preparations of peripheral blood mononuclear cells (PBMC). NK cells are intrinsically unreactive to foreign major histocompatibility (MHC) molecules. This has made them an attractive alternative for generation of therapeutic cell products, as their insensitivity to antigens presented by MHC allows them to be used in an allogenic context with minimal risk of graft versus host disease (GvHD). Although NK cells carry activating and inhibitory receptors for MHC molecules, the MHC mismatch between graft and host is usually not sufficient to contribute to pathology (1). Recent research indicates, however, that host-specific factors may be important to optimize their potential (2). Furthermore, improvements increasing homing to sites of tumor growth such as bone marrow or to the tumor itself by enhanced chemokine receptors have shown promise (3, 4).

**Figure 1:**
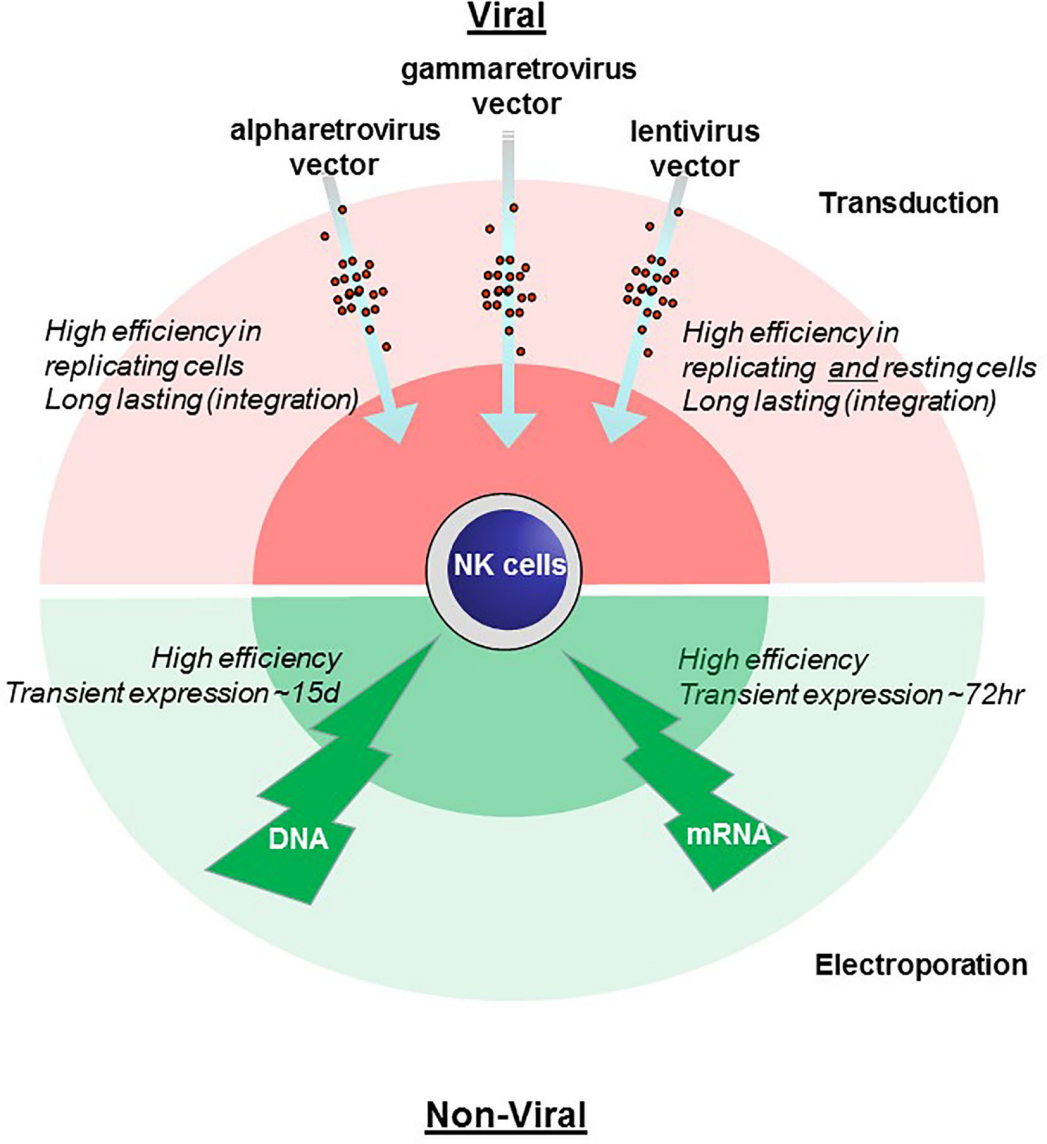
Comparison of methods for genetic engineering of NK cells. Viral transduction (pink, upper) and non-viral electroporation (green, lower) methods of gene engineering NK cells are illustrated with advantages and disadvantages noted.

Chimeric antigen receptors (CARs) are receptor proteins that have been engineered to allow specific recognition of a target protein and induction of secondary signaling. The recognition domain is usually derived from the antigen-binding regions of an antibody. This is presented on the cell surface by a so-called hinge or spacer region providing flexibility. This is then bound by a transmembrane domain to intracellular signaling domains, usually CD3-zeta. Costimulatory domains are also often included intracellularly to enhance signaling and longevity post activation. CARs have mostly been used in T cells to retarget them against tumors. The CAR uses the existing T cell cytotoxic machinery to destroy the targeted malignant cells. The fact that other cell types are also known to attack tumor cells prompted recent interest in CAR expression in non–T cells. The most notable success has been the Natural Killer (NK) cell. NK cells were first identified by their unique ability to kill tumor cells without prior antigen priming. NK cells with CAR receptors (CAR-NK) have now been recognized as a potent tool against cancer (5–8). Initially, the creation of CAR-NK cells was undertaken with constructs developed for CAR-T cells; however, recent developments have incorporated adaptions to NK cells. Nevertheless, NK cells pose special and unique problems. Unlike T cells and B cells, NK cells are not typically clonally expanded, making the generation, maintenance and expansion of CAR-NK cells challenging. The most important stage in the generation of NK-CAR cells is the introduction of the genetic element into the NK cell itself (**Figure 1**) and the subsequent expansion of the CAR-NK cells.

## Sources of Natural Killer Cells

Peripheral-blood derived NK cells can be readily isolated from peripheral blood but are difficult to engineer. The reasons behind this are unclear but include low transduction efficiency combined with poor expansion. In order to avoid this bottleneck NK cells have been generated from induced pluripotent stem cells (iPSC), which can yield many more cells and are more permissive to engineering (9). NK cells can also be generated from umbilical cord blood. NK cells from this source are more readily engineered due to their higher proliferative capacity, as was recently demonstrated in the first published clinical trial of CAR-NK cells (10). Despite this success, a possible disadvantage is the relatively immature nature of cord blood derived NK cells. Several trials have used NK cell lines, particularly cell line NK-92 (8). Cell lines are relatively easy to engineer but are undesirable both due to safety considerations and as they must be lethally irradiated before administration, so they cannot persist in the host and cannot therefore give long-term protection. Recently, feeder cell lines have come into common use to expand NK cells *ex vivo*. These MHC-negative cells lines, in particular K562, are often engineered to express membrane-bound cytokines (IL-15 and IL-21) and are irradiated before being used (11).

## Non-Viral CAR-Natural Killer Cell Engineering: mRNA and DNA Transfection

In contrast to gene expression *via* viral vectors CAR expression with non-viral–based methods is usually transient, being present for a few days (12). Although long-term expression can be achieved if the sequence can integrate, as is the case with transposon-based systems (13). Typically, nucleic acid introduction is achieved by electroporation, which is a simple and cost-effective method and therefore appropriate for large-scale clinical applications. A major disadvantage is that the permeabilization of the cell membrane by electric pulses can easily result in high cell death rates by unregulated exchange of interior or exterior cell components or the creation of permanent membrane leakage (14).

## mRNA Transfection of Natural Killer Cells

Primary CAR-NK generation by mRNA electroporation was initially investigated in 2010 by using an anti-CD19 transgene for transfection in unstimulated and expanded NK cells (15). Expression of the CAR ranged from 33% to 81%. It has been determined that for clinical products expression of a CAR after electroporation is dependent on the dose of mRNA (25–200 µg/ml) and increases the more nucleic acid is applied (16). Interestingly, the viability of expanded cells after one day post-transfection was lower (54%) than for unexpanded cells (64%) (15). However, other groups were not able to transfect peripheral blood or cord blood derived NK cells at transfection rates higher than 10% even with IL-2 stimulation (17). In contrast transfection efficiency for the NK-92 cell line with mRNA encoding an anti-CD19 CAR resulted in higher yields (47.2 ± 8%) (18). More recent protocols have achieved efficient expression of a duration of at least 72 h (3, 19). Recently it has been shown that the co-expression of two transgenes, one being a CAR and the other a chemokine receptor is possible using mRNA electroporation. This yields a transfection rate of 90% modified cells (3). Typically, 5 µg of capped, polyadenylated mRNA is electroporated in a 2-mm cuvette using a voltage of 240–500 V for 4–5 ms (3, 19).

The transfer of mRNA transfection into the clinic has been aided by a recently published protocol for large-scale NK cell expansion in compliance with current Good Manufacturing Practice (cGMP) (20). Large-scale cGMP-compliant electroporation systems such as MaxCyte or CliniMacs are now available for the generation of therapeutic product.

The introduction of CAR mRNA into resting NK cells using a chemical method (charge-altering releasable transporters, or CARTs) has been recently reported (21). This method combined both a higher efficiency compared to electroporation using the 4D nucleofector device and caused less damage and phenotypic change. Finally, CAR-NK cells derived were cytotoxic to CD19+ target cells.

In summary, mRNA transfection is highly efficient but with significant drawbacks. Transfection of complex multigene constructs often necessitates cotransfection of separate mRNA due in part to the difficulty in generating long mRNA sequences. Furthermore, despite its efficiency mRNA is inherently labile, being non-integrative and non-replicative, resulting in a very short period of expression.

## DNA Transfection of Natural Killer Cells

Initially, electroporation with DNA was reported successfully for the cell line NK-92 (18) but not for freshly isolated or expanded human NK cells. In a recently published protocol, however, the authors were able to transfect IL-2 expanded primary NK cells by prior optimization of plasmid DNA concentration, target cell number, plasmid size, buffer conditions, voltage, and number and width of pulses (22). Each optimization step contributed to efficiency of transfection with no single element dominating. In contrast, for resting NK cells cell number was of paramount importance with 2–6 × 10^7^ cells/ml being the optimal range. After using the new protocol to transfect a first- and second- generation CAR into IL-2 expanded NK cells, 40% transfected cells were observed with a cell viability to up ~60%. This represents a 5-fold increase in efficiency over standard protocols (22). After DNA electroporation the viability of cells is lower compared to mRNA electroporation, probably due to the harsher transfection conditions needed for DNA to reach the NK cell nucleus (18) or due to activation of the innate immune system. Transfected DNA is more durable than mRNA, with expression persisting up to 15 days (23).

To summarize, DNA transfection is less efficient, however complex constructs can be readily introduced and like mRNA non-integrated DNA is self-limiting which thus gives a favorable safety profile compared to viral methods (22).

## Transduction of Natural Killer Cells: Viral Vectors

Transduction refers to the introduction of genetic material *via* viral vectors, including the retroviral and lentiviral-based vectors (24). During the life-cycle of retroviruses viral RNA is reverse transcribed into double-stranded cDNA which is then semi-randomly integrated into the genome of the infected cell (25). For these reasons, this strategy typically takes longer until the gene is expressed. Vectors based on these pathogens have several advantages making it relatively simple to create complex vectors and subsequently reliably introduce them into cells. Typically, these vectors can be up to 10 kb in size without incurring significant loss of titer during production, allowing inserts of up to 7–8 kbp. Furthermore, as these vectors integrate, this allows the permanent modification of the cell in the absence of antibiotic resistance markers. The modified cells can then be maintained in the host over long periods of time.

The sensitivity of NK cells to foreign genetic material and the stressful process of transduction typically results in low levels of transduction and high apoptosis. Efficiency in transfecting NK cells is therefore relatively low compared to T-cells. This is due to resistance to viral transduction from innate defense mechanisms guided by pattern recognition receptors recognizing foreign genetic material (26, 27). In order to prevent this inhibitory chemicals such as BX795, which inhibits PDK1, can be added during transduction. This blocks activation of signaling pathways mediated by RIG-I like receptors or Toll-like receptor 3 (28). It has been demonstrated that lentiviral transduction efficiency can be enhanced ~4 fold by this strategy (29). Nevertheless, it is sometimes necessary to use multiple rounds of transduction to achieve an adequate transgene expression (30).

In order to increase the safety profile of viral vectors, expression systems have been created where the envelope protein is expressed on a separate plasmid. This allows safe generation of modified viral particles with exogenous envelope proteins, in a process termed pseudotyping. One way of enhancing transduction of NK cells is the selection of the best pseudotyping envelope protein. The commonly used vesicular stomatitis virus (VSV) glycoprotein G is usually highly efficient, as it binds to the LDL receptor which is present in a wide variety of cells (31). However, it is inefficient for infection of lymphoid cells, requiring high viral titers which in turn are often toxic to the cells. The choice of envelope proteins from lymphotropic viruses such as measles or baboon retrovirus, however, has been shown to improve both transduction and integration (32). Successful transduction also depends on diffusion of the virus to the cell surface and adsorption into the target. Enveloped viral particles are typically negatively charged as they derive from the cell membrane. This results in repulsion of virion and cell, interfering with transduction. Cationic polymers such as hexadimethrine bromide (polybrene) or protamine sulfate are used to neutralize the negative charge of the virion, allowing better adsorption efficiency and membrane fusion (33). An alternative strategy is to enhance colocalization of cells and virus by using crosslinking agents such as Retronectin or Vectofusin-1. These have been reported to outperform their cationic polymer counterparts (17, 34). Retronectin, a chimeric peptide derived from fibronectin, promotes the interaction and colocalization between virus particles and counterparts on the cell surface (34). Similarly, Vectofusin-1 facilitates the adhesion and the fusion of the virus with the cellular plasma membrane, although the concrete mechanism remains unclear (35). There is no consensus on which enhancer shows greater optimization of transduction (35, 36).

## Retroviral Vectors

Vectors generated from the related alpharetroviral and gammaretroviral viruses have been used to transduce primary lymphatic cells, including NK cells (30, 37). A significant limitation for retroviral transduction is that retroviral cDNA can only integrate into the NK cell genome during mitosis when the nuclear membrane dissolves. This requirement is particularly problematic in non-replicating primary cells, but less so for activated NK cell lines (38).

## Lentiviral Vectors

Lentiviral vectors are considered genetically more complex and in contrast to their retroviral counterparts can integrate their genetic information into non-dividing cells (39). Recent data shows that the performance of lentiviral vectors in generating CAR-NK cells depends on the envelope protein they express. For the commonly used VSV-G envelope proteins the highest transduction efficiency of primary NK cells using VSV-G pseudotyped particles was found with lentiviral vectors compared to retroviral vectors (34). Which envelope protein has the best performance is unclear as the transduction efficiency for lentiviral vectors pseudotyped with VSV-G or feline endogenous retrovirus envelope protein RD114-TR was similar for primary human NK cells (34). Another group found that a lentiviral/VSV-G vector produced less CD19-CAR expressing cells compared to a RD114-TR pseudotyped lentiviral vector (36). Furthermore, a Baboon envelope pseudotyped lentiviral vector BaEV-LV was significantly better than both the RD-114-TR as well as VSV-G pseudotyped lentiviral vector (32). VSG-V binds to the low density lipid receptor LDL-R which is poorly expressed on activated NK cells (5). In contrast, RD114 binds to the sodium-dependent neutral amino acid transporter ASCT-1 and ASCT-2, as does the BaEV envelope protein (40–42). The ASCT-1 transporter is strongly expressed on NK cells and ASCT-2 is upregulated after activation of NK cells with IL-2 and IL-15 (5). The BaEV envelope protein has also been shown to bind to the glycosylated form of ASCT-1 in contrast to RD-114-TR. This may explain its greater efficiency in transduction (42).

It has been reported that lentiviral transduction can be further optimized by using spinfection, a method where centrifugation at a low RPM is applied. Spinfection transduction rates range from 19% to 73% with CD19 CAR lentiviral transduced cord blood derived NK cells, compared to a range from 12% to 30% for static transduction (17).

In terms of optimization for transduction protocols Müller et al. compared alpharetroviral vectors with lentiviral vectors. A RD114-TR pseudotyped alpharetroviral vector could achieve 82.9% of NKs transduced with CD19 CAR using Vectofusin-1–based transduction (36). A similar comparative study of viral vectors from Suerth et al. confirms the superiority in transfection efficiency when a RD114-TR pseudotyped alpharetroviral vector was used. Although transduction was performed with Rectofusin both transduction enhancers reliably optimized transduction efficiency (34). It is thought that Vectofusin-1 might be beneficial for large-scale expansion due to a simpler usage (36). Because of the stable high rate of transgene expression by using a RD114-TR alpharetroviral vector Kellner et al. established a protocol for production of genetically modified NK cells compliant with GMP. Following the instructions, transduction procedure resulted in >90% CAR transduced cells (43). In order to achieve this efficiency proliferating NK cells were transduced using retronectin. A possible drawback of this method, however, is the use of the K-562 cell line to expand the NK cells both prior and post transduction. Furthermore, a relatively simple CAR was used to transduce (43). In conclusion, there is no currently available gene transfer method that is universally applicable. All have advantages and drawbacks which we have illustrated in **Figure 1**.

## Outlook

Several novel methods to introduce genes into NK cells are under development. These include alternative viral vectors with a higher safety profile such as adenovirus associated virus (AAV) vectors. Advances in mRNA generation and electroporation technology will also bring improved transfection. Combining the two methods, for example with transposons or with CRISPR/Cas9-based integration, would combine long term expression with the efficiency of electroporation. Transposon technology in particular offers integration without complex, expensive, and potentially dangerous viral transduction systems. One disadvantage that remains with this system is its relative inefficiency. Finally, improvement in primary NK expansion protocols will also bring an improvement in gene engineering, as healthy proliferating cells are more readily engineered. The vectors themselves will also be become optimized to the NK microenvironment, for example by using DAP12 or NKG2D signaling domains. Which construct is best suited for NK cells is a focus of current research.

The durability of the CAR-NK cells within the host is also currently a matter of debate. CAR-T cells can persist in the host for years and there is clear data showing that this contributes to tumor clearance. Whether this is the case for CAR-NK cells is unclear. It is currently accepted that allogenic NK cells have a relatively short half-life (44), although persistence of CAR-NK cells *in vivo* for at least 1 year has been recently reported (10). Long-term follow up of clinical trials of primary NK cells will elucidate if persistence of CAR-NK cells contributes to therapeutic efficacy.

## Concluding Remarks

Although NK cells have many desirable characteristics, there remain significant problems in the production of CAR-NK cells for therapeutic purposes. The introduction of foreign genetic material and subsequent expansion of the NK cells is difficult, making the development of feasible and reproducible GMP protocols a challenge. The viability of the CAR-NK cells is central to the success of the therapeutic product, as the long-term persistence of tumor-specific CAR cells in the host is thought to promote the therapeutic efficacy.

Currently, the most successful alternatives for introducing genes into NK cells are either rapid transient expression by electroporation or slow sustained expression by viral vectors. The correct choice of transfection protocol is thus an important element in the design and execution of a successful clinical trial.

## Author Contributions

PS, MR, and GP reviewed papers and wrote the paper. All authors contributed to the article and approved the submitted version.

## Conflict of Interest

The authors declare that the research was conducted in the absence of any commercial or financial relationships that could be construed as a potential conflict of interest.
